# *Camellia sinensis* Prevents Perinatal Nicotine-Induced Neurobehavioral Alterations, Tissue Injury, and Oxidative Stress in Male and Female Mice Newborns

**DOI:** 10.1155/2017/5985219

**Published:** 2017-05-15

**Authors:** Jamaan S. Ajarem, Gadh Al-Basher, Ahmed A. Allam, Ayman M. Mahmoud

**Affiliations:** ^1^Department of Zoology, Faculty of Science, King Saud University, Riyadh, Saudi Arabia; ^2^Department of Zoology, Faculty of Science, Beni-Suef University, Beni-Suef, Egypt; ^3^Physiology Division, Department of Zoology, Faculty of Science, Beni-Suef University, Beni-Suef, Egypt

## Abstract

Nicotine exposure during pregnancy induces oxidative stress and leads to behavioral alterations in early childhood and young adulthood. The current study aimed to investigate the possible protective effects of green tea (*Camellia sinensis*) against perinatal nicotine-induced behavioral alterations and oxidative stress in mice newborns. Pregnant mice received 50 mg/kg *C. sinensis* on gestational day 1 (PD1) to postnatal day 15 (D15) and were subcutaneously injected with 0.25 mg/kg nicotine from PD12 to D15. Nicotine-exposed newborns showed significant delay in eye opening and hair appearance and declined body weight at birth and at D21. Nicotine induced neuromotor alterations in both male and female newborns evidenced by the suppressed righting, rotating, and cliff avoidance reflexes. Nicotine-exposed newborns exhibited declined memory, learning, and equilibrium capabilities, as well as marked anxiety behavior. *C. sinensis* significantly improved the physical development, neuromotor maturation, and behavioral performance in nicotine-exposed male and female newborns. In addition, *C. sinensis* prevented nicotine-induced tissue injury and lipid peroxidation and enhanced antioxidant defenses in the cerebellum and medulla oblongata of male and female newborns. In conclusion, this study shows that *C. sinensis* confers protective effects against perinatal nicotine-induced neurobehavioral alterations, tissue injury, and oxidative stress in mice newborns.

## 1. Introduction

Maternal smoking during pregnancy is a versatile risk factor that represents a public health concern [1]. Adverse perinatal outcomes and health complications, including respiratory disorders and childhood cancers, are associated with exposure to smoking in utero [2–4]. Additionally, low birth weight, premature birth, neonatal death, neural tube defects, and congenital anomalies have been identified as adverse effects of maternal smoking [5, 6].

Nicotine and its metabolite cotinine can readily cross the placenta and bind to the nicotinic acetylcholine receptors [7]. These receptors are known to be expressed by the second gestational week in rodents and in the first trimester in humans [8, 9]. The study of Berlin et al. [10] showed that cotinine concentrations in newborn's cord blood are similar to that of their smoking mothers. Through binding to these receptors, nicotine disturbs the cholinergic system, brain development, neuronal migration, synaptogenesis, and neurotransmitter release [11]. These nicotine-induced adverse effects can alter fetal brain development and produce neurobehavioral impairments later in life [1].

In this context, studies have demonstrated that maternal smoking during pregnancy leads to behavioral alterations in early childhood and can extend to young adulthood [12, 13]. Experimental animals exposed to tobacco alkaloid extracts showed delayed development of several behavioral patterns [14]. Nicotine exposure in utero resulted in memory and learning [15, 16] and sensory processing defects [17] in rodents. In clinical settings, newborns exposed to nicotine in utero exhibited poor attention, increased tremors, hypertonicity, startle responses, irritability, and deficient speech processing [18–21]. Newborns also showed an attenuated response to auditory stimuli [20, 22]. This effect, later in life, can contribute to language and learning impairments [20, 22]. In addition, exposed infants showed poor self-regulation within the first month of life [23]. Thus, continuous exposure to nicotine during fetal development in utero and early developmental period of the pups after birth has bad impacts on the developing brain tissues and neurobehavioral and cognitive functions.

Excessive production of reactive oxygen species (ROS) plays a key role in nicotine-induced neurodevelopmental alterations [24]. Nicotine has been reported to induce oxidative stress both in vivo [25, 26] and in vitro [27]. Also, nicotine-induced cell death in various brain regions and memory impairment have been attributed to excessive ROS production [28–30].

Based on the previous findings, counteracting oxidative stress could represent an effective strategy to protect against nicotine-induced alterations in newborns. Therefore, the current study aimed to demonstrate the possible protective effects of green tea (*Camellia sinensis*) extract against perinatal nicotine-induced neurodevelopmental and behavioral alterations and oxidative stress in mice newborns. *C. sinensis* and its bioactive polyphenols are well-known to possess potent antioxidant and radical scavenging efficacies [31].

## 2. Materials and Methods

### 2.1. Preparation of *C. sinensis* Extract

Fresh leaves of *C. sinensis* were purchased from a local herbalist and ground to a powder using an electric grinder. Fifty g of the fine powder was added to one liter of boiled water and left for 15 min. The infusion was then filtered and freshly used.

### 2.2. Experimental Animals and Treatments

All the experimental protocols and investigations were approved and complied with the *Guide for Care and Use of Laboratory Animals* published by the US National Institutes of Health (NIH Publication Number 85–23, revised 1996) and was approved by the Ethics Committee for Animal Experimentation at King Saud University. Twenty-eight females and 14 males of Swiss Webster mice (*Mus musculus*) of 10–12 weeks were used in this study. The mice were obtained from the animal house of the College of Pharmacy, King Saud University, Riyadh, Saudi Arabia. The animals were housed in a well-ventilated animal's room in standard mice cages at a room temperature around 25°C and 12 h light/dark cycle. Standard rodent diet and water were supplemented ad libitum. The estrous cycle of females was estimated and each 3 proestrous females were housed with a male in a standard rodent mating cage for 12 h. The appearance of the vaginal plug in the morning was considered the first day of pregnancy. After mating, each dam has been incubated single in a cage where it will incubate its newborns after delivery. The date of birth for each dam was recorded. The number of newborns for each mother was fixed to be eight. The dates of appearance and development of external features such as eye opening, fur appearing, and body weights were recorded. The newborns were exposed to some behavioral investigations.

The mice were randomly divided into 4 groups; each comprises 4-5 mothers as following:

*Group 1 (Control)*. Pregnant mice received distilled water by oral gavage from the first day of pregnancy (PD1) until the 15th day after birth (D15) and subcutaneously injected with physiological saline from the 12th day of pregnancy (PD12) until D15 after birth.

*Group 2 (C. sinensis)*. Pregnant mice received 50 mg/kg body weight *C. sinensis* extract [32] by oral gavage from PD1 until D15 and subcutaneously injected with saline from the PD12 until D15.

*Group 3 (Nicotine)*. Pregnant mice received distilled water by oral gavage from PD1 until D15, and subcutaneously injected with 0.25 mg/kg body weight nicotine (SOMATCO, Riyadh, KSA) [33] dissolved in saline from PD12 until D15.

*Group 4 (Nicotine + C. sinensis)*. Pregnant mice received 50 mg/kg body weight *C. sinensis* extract by oral gavage from PD1 until D15 and subcutaneously injected with 0.25 mg/kg body weight nicotine (SOMATCO, Riyadh, KSA) dissolved in saline from PD12 until D15.

### 2.3. Behavioral Study

#### 2.3.1. Righting Reflex

This reflex was conduct according to Ajarem and Ahmad [34] where the newborns examined at postnatal days (D) 1, 5, 10, 15, and 20 for male and female newborns by placing the newborn on its back. The time consumed till righting on its four limbs was measured and recorded. The response is negative when the righting time duration exceed 120 seconds.

#### 2.3.2. Rotating Reflex

This reflex was conducted according to Ajarem and Ahmad [35]. Each animal was placed on the inclined surface at an angle of 30 degrees and the direction of its head down and was being monitored to be moving its body in the opposite direction and the time spent was recorded. This reflex was examined at D1, D5, D10, D15, and D20; the maximum duration of this test is 120 seconds.

#### 2.3.3. Cliff Avoidance Reflex

The newborns (males and females) were placed on the edge of a wood piece above the ground, and then, the time spent till turning the back in opposite direction by half of circle at 180 degrees angle was recorded [36].

#### 2.3.4. Locomotion Activity Reflex

This test was conducted for male and female newborns at D22 in the locomotory box as mentioned by Ajarem and Ahmad [37]. The newborns were examined for all activities such as number of squares crossed, number of rears, number of wall rears, number of cleaning, duration of locomotion, and duration of immobility.

#### 2.3.5. Fear and Anxiety Reflex

This reflex was conducted at D25 for male and female newborns using the elevated perpendicular plus maze according to Abu-Taweel [38]. The newborns were placed in the middle of the maze at the intersection of the arms point facing the open arm. The examination time was 300 seconds with recording of all movements and activities, including the number of entries into the arms and center, and the time spent in the arms and center of the maze.

#### 2.3.6. Equilibrium Reflex

This reflex was conducted at D30 using a rotarod instrument (Ugo Basile, Italy) as previously mentioned by Allam et al. [39]. The reflex measures the balance ability in the investigated mice. Each animal was placed on a horizontal rod which rotates slowly at 1 cycle/sec. The newborns will try to stay on the rod as more as they can, but finally, they fall. The instrument records the time which the animal spends on the rod. This time reflect the ability of the newborn to resist against falling.

#### 2.3.7. Active Avoidance Reflex (Shuttle-Box Test)

This reflex measures the memory and learning ability for the newborns and conducted according to the method described by Abu-Taweel et al. [40] using the shuttle-box-automated reflex conditioner (Ugo Basile, Italy). The newborns were investigated at D35, and the results were automatically recorded by the instrument. Each animal was exposed to the test for 30 trials. The lamp and the bell will be operated thirty times, and the electricity shocks depending on the animal's ability to learn. The healthy animals (who learn quickly) move from one room to another on seeing light of the lamp and hearing the bell directly to prevent stun electricity. The test begins by placing the animal in one of the two rooms and leaving it while it explores the place, then the actual test starts, which ends with the thirtieth over again, and this period can be less than the animal's ability to learn (to escape when it sees the lamp and hears the bell).

### 2.4. Biochemical and Histological Study

At D7, D15, and D30 after birth, 6 pups from each group were sacrificed by decapitation, and samples were collected. Samples from the cerebellum and medulla oblongata were homogenized in cold phosphate-buffered saline and used for assaying lipid peroxidation [41], reduced glutathione (GSH) [42], and superoxide dismutase (SOD) activity [43]. Other samples from the cerebellum and medulla oblongata were fixed in neutral-buffered formalin and processed for staining with hematoxylin and eosin.

### 2.5. Statistical Analysis

The data were analyzed by one- or two-way ANOVA followed by Tukey's test post hoc analysis using GraphPad Prism version 5 (San Diego, CA, USA). The obtained results were presented as mean ± standard error (SEM) with a *P* value less than 0.05 being considered significant.

## 3. Results

### 3.1. Effect of *C. sinensis* on Body Weight, Hair Appearance, and Eye Opening in Control and Nicotine-Induced Mice Newborns

**Figure 1:**
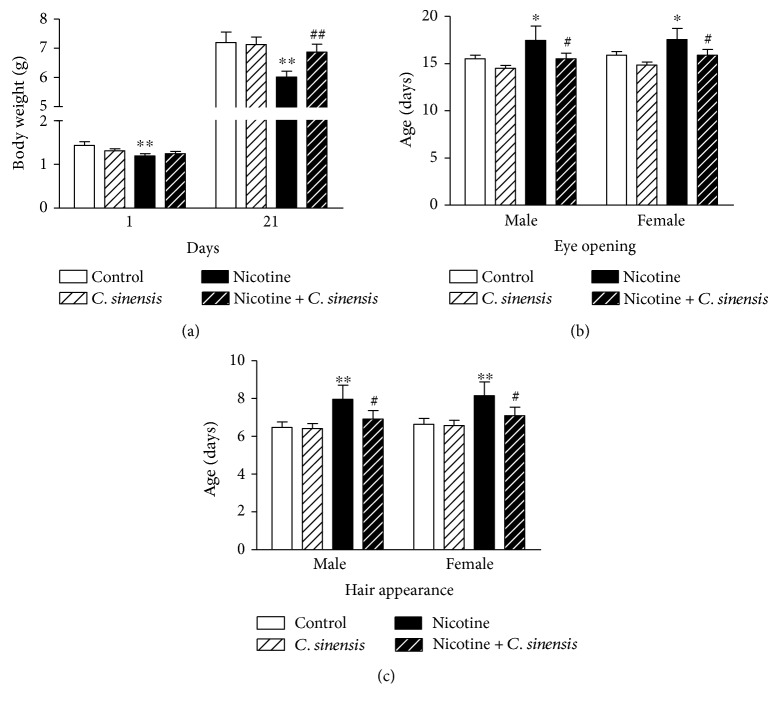
Effect of *C. sinensis* on (a) body weight, (b) hair appearance, and (c) eye opening in control and nicotine-induced mice newborns. Data are M ± SEM. ^∗^*P* < 0.05 and ^∗∗^*P* < 0.01 versus control. ^#^*P* < 0.05 and ^##^*P* < 0.01 versus nicotine.

Body weight, eye opening, and hair appearance were determined as physical assessments during the weaning period. Body weight of the nicotine-induced mice newborns showed a significant (*P* < 0.01) decrease at D1 and D21 after birth when compared with that of the control group (Figure 1(a)). *C. sinensis* supplementation produced a nonsignificant (*P* > 0.05) effect on body weight of either control or nicotine-exposed mice offspring at D1. Supplementation of *C. sinensis* to nicotine-exposed pregnant mice significantly (*P* < 0.01) improved body weight of the newborns at D21 after birth. Similar effects were recorded in both male and female newborns.

*C. sinensis* supplementation produced nonsignificant (*P* > 0.05) effects on eye opening in both male and female mice offspring (Figure 1(b)). Both male and female nicotine-induced mice newborns showed a significant (*P* < 0.05) delay in eye opening when compared with the control groups. *C. sinensis* administration significantly (*P* < 0.05) prevented the nicotine-induced delay in eye opening in both genders.

Male and female mice born to nicotine-induced mothers showed a significant (*P* < 0.01) delay in hair appearance. Treatment of the nicotine-induced mice with *C. sinensis* significantly (*P* < 0.05) prevented the delay in hair appearance in both male and female newborns (Figure 1(c)). *C. sinensis* supplementation exerted nonsignificant (*P* > 0.05) effect on hair appearance time in either male or female newborns.

### 3.2. *C. sinensis* Prevents Nicotine-Induced Neurobehavioral Alterations in Mice Newborns

#### 3.2.1. Neuromotor Maturation

To evaluate the effect of nicotine on the maturation of neuromotor reflexes and the protective efficacy of *C. sinensis*, the righting, rotating, and cliff avoidance reflexes were determined at D1, D5, D10, D15, and D20 after birth.

**Figure 2:**
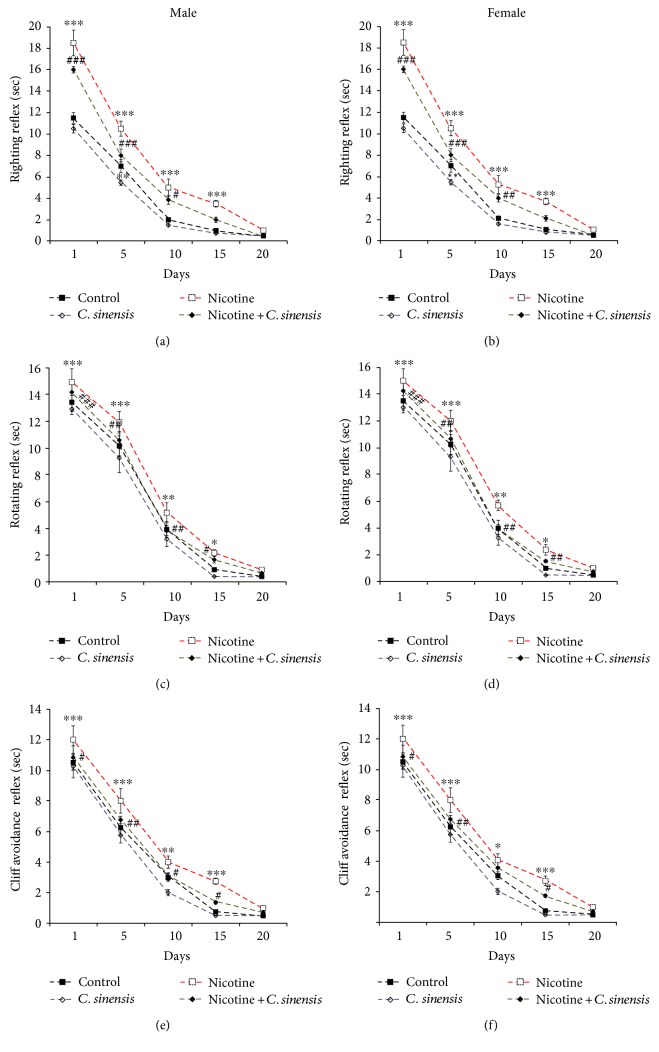
*C. sinensis* prevents nicotine-induced suppression of (a-b) righting, (c-d) rotating, and (e-f) cliff avoidance reflexes in mice newborns. Data are M ± SEM. ^∗^*P* < 0.05, ^∗∗^*P* < 0.01, and ^∗∗∗^*P* < 0.001 versus control. ^#^*P* < 0.05, ^##^*P* < 0.01, and ^###^*P* < 0.001 versus nicotine.

The righting reflex was significantly (*P* < 0.001) suppressed at D1, D5, D10, and D15 after birth in nicotine-exposed male (Figure 2(a)) and female (Figure 2(b)) newborn mice when compared with the corresponding controls. *C. sinensis* supplementation significantly (*P* < 0.001) improved the righting reflex at D1 and D5 in male and female newborns. At D15, the righting reflex showed significant amelioration in *C. sinensis*-treated male (*P* < 0.05) and female (*P* < 0.01) nicotine-induced newborns.

The rotating reflex showed a similar pattern where nicotine-exposed mice newborns showed reduced performance of the rotating reflex at D1 (*P* < 0.001), D5 (*P* < 0.001), D10 (*P* < 0.01), and D15 (*P* < 0.05) after birth. Treatment of the nicotine-exposed mice with *C. sinensis* significantly improved the rotating reflex throughout the weaning period in both male (Figure 2(c)) and female (Figure 2(d)) offspring.

Regarding the cliff avoidance, male nicotine-exposed offspring showed significantly declined reflex at D1 (*P* < 0.001), D5 (*P* < 0.001), D10 (*P* < 0.01), and D15 (*P* < 0.001) after birth (Figure 2(e)). Similarly, the nicotine-exposed female newborn exhibited significant suppression in the cliff avoidance reflex at D1 (*P* < 0.001), D5 (*P* < 0.001), D10 (*P* < 0.05), and D15 (*P* < 0.001) after birth (Figure 2(f)). Treatment of the nicotine-exposed mice with *C. sinensis* significantly improved the cliff avoidance reflex in both male and female offspring.

Of note, supplementation of *C. sinensis* did not induce any significant changes in the neuromotor reflexes of the control group.

#### 3.2.2. Active Avoidance Test

The effect of *C. sinensis* supplementation on memory and learning ability in control and nicotine-induced newborns was measured using the shuttle-box test.

**Figure 3:**
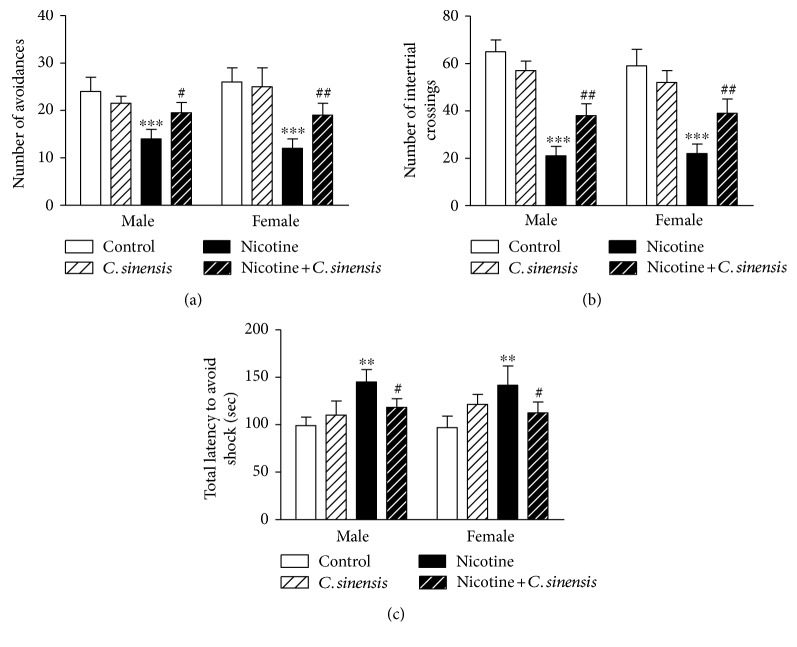
*C. sinensis* improves active avoidance responses in nicotine-exposed mice newborns. Data are M ± SEM. ^∗∗^*P* < 0.01 and ^∗∗∗^*P* < 0.001 versus control. ^#^*P* < 0.05 and ^##^*P* < 0.01 versus nicotine.

Nicotine significantly (*P* < 0.001) decreased the number of avoidances during the trial period in both male and female offspring when compared with that in the corresponding controls (Figure 3(a)). *C. sinensis* supplementation improved the number of avoidances in nicotine-induced both male (*P* < 0.05) and female (*P* < 0.01) mice offspring, with no effect on control mice.

Nicotine-induced male and female newborns showed a significant (*P* < 0.001) decrease in the number of intertrial crossings between the chambers in the absence of shock, an effect that was significantly (*P* < 0.01) ameliorated following treatment with *C. sinensis* extract (Figure 3(b)). *C. sinensis* produced a nonsignificant (*P* > 0.05) effect on the number of intertrial crossings when supplemented to control mice.

The total time taken to avoid the shock during the entire trials was measured. As depicted in Figure 3(c), nicotine-induced both male and female mice offspring were poor learners and took significantly (*P* < 0.01) longer time in avoiding the shock when compared with the control group. Supplementation of *C. sinensis* extract markedly (*P* < 0.05) improved the learning ability of nicotine-induced male and female mice newborn, with no effect on the learning ability of control mice.

#### 3.2.3. Locomotor Activity Test

Nicotine-induced weaned male mice showed significant decrease in the number of squares crossed (*P* < 0.001), wall rears (*P* < 0.05), and locomotion duration (*P* < 0.001) when compared with the control group (Table 1). On the other hand, the number of washes and the immobility duration were significantly increased in the nicotine-induced animals. *C. sinensis* supplementation produced a marked amelioration in all elements of the locomotor activity in both control and nicotine-induced weaned mice.

In female-weaned nicotine-induced mice, the number of squares crossed, wall rears, and locomotion duration were significantly (*P* < 0.001) decreased with a concomitant significant (*P* < 0.01) increase in the immobility duration (Table 1). However increased, the number of washes showed a nonsignificant (*P* > 0.05) change in nicotine-induced female weaned mice when compared with the corresponding control group. *C. sinensis* supplementation significantly improved the locomotor activity in both control and nicotine-induced female mice.

#### 3.2.4. Equilibrium Reflex

**Figure 4:**
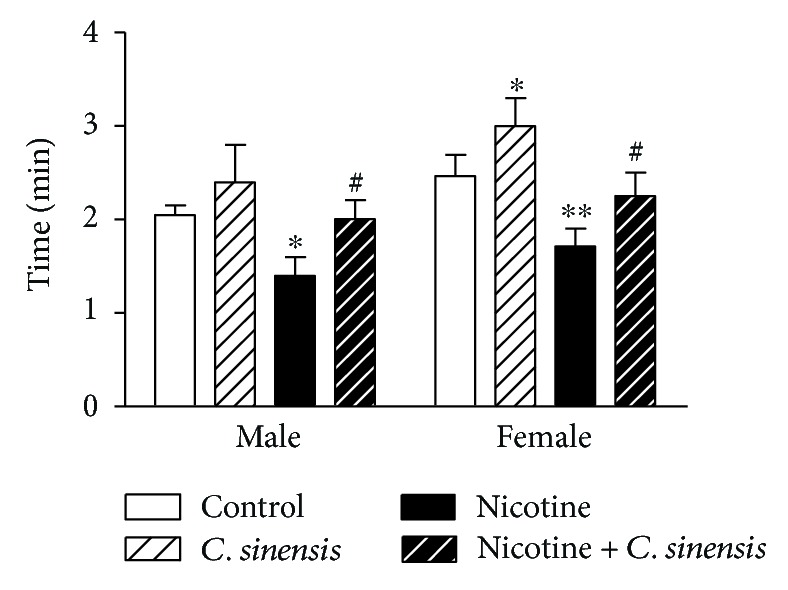
*C. sinensis* alleviates equilibrium reflex in nicotine-exposed mice newborns. Data are M ± SEM. ^∗^*P* < 0.05 and ^∗∗^*P* < 0.01 versus control. ^#^*P* < 0.05 versus nicotine.

In the rotarod test, *C. sinensis* significantly (*P* > 0.05) increased the time that the mice spent on the rotating rod in female but not in male animals (Figure 4). Nicotine administered during the pregnancy period significantly affected the balance ability of the animals as evidenced by the decreased time spent on the rotating rod by both male (*P* < 0.05) and female (*P* < 0.01) mice. *C. sinensis* extract produced a significant (*P* < 0.05) improvement in the balance ability of nicotine-induced both male and female mice.

#### 3.2.5. Anxiety Behavior in the Elevated Plus-Maze Test

**Figure 5:**
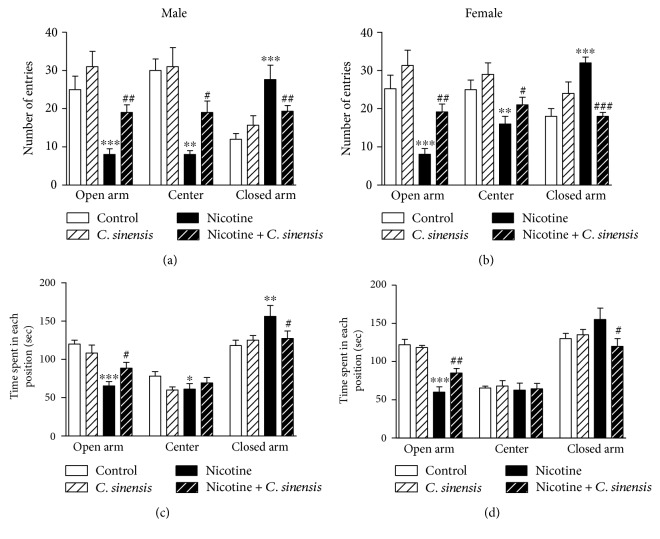
Protective effect of *C. sinensis* against nicotine-induced anxiety in mice newborns. Data are M ± SEM. ^∗^*P* < 0.05, ^∗∗^*P* < 0.01, and ^∗∗∗^*P* < 0.001 versus control. ^#^*P* < 0.05, ^##^*P* < 0.01, and ^###^*P* < 0.001 versus nicotine.

The elevated plus maze is frequently used to evaluate the anxiety-like behavior in animal models [44]. In the present study, administration of nicotine during gestation significantly reduced the number of entries and the time spent to explore the open arm (*P* < 0.001) in both male (Figure 5(a)) and female (Figure 5(b)) newborn mice. *C. sinensis* significantly (*P* < 0.01) increased the number of entries to explore the open arm. Similarly, the time spent to explore the open arm was significantly increased in nicotine-induced male (Figure 5(c); *P* < 0.05) as well as female (Figure 5(d); *P* < 0.01) mice newborns. *C. sinensis* affected neither the number of entries nor the time spent to explore the open arm when supplemented to control mice.

On the contrary, nicotine administration during pregnancy significantly increased the number of entries and the time spent in the closed arm in both male and female mice newborn. *C. sinensis* supplementation significantly decreased the number of entries and time spent in the closed arm in nicotine-induced both male and female mice.

### 3.3. *C. sinensis* Prevents Nicotine-Induced Histological Alterations in the Cerebellum and Medulla Oblongata of Mice Newborns

**Figure 6:**
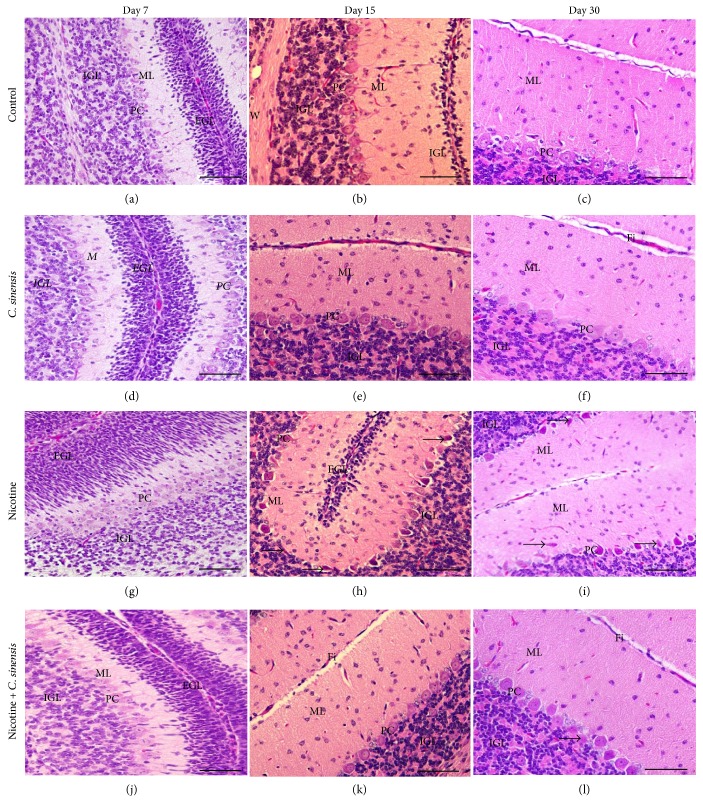
Sagittal sections in the cerebellum of (a–c) control newborns showing wide external granular layer (EGL) at day 7 which become thin at day 15 and disappeared at day 30. The molecular layer (ML) is defined at day 7, wide at day 15, and incubated by mature neurons at day 30. The Purkinje cells (PC) appear arranged in one row at day 7 and become more developed and mature at day 15 and day 30. The internal granular layer (IGL) received the migrated cells from the external granular layer, so it appears condensed at the three investigated ages. (d–f) The *C. sinensis*-administered group showing well-developed cerebellar fold layers similar to those of normal newborns. (g–i) The nicotine-exposed group showing a delay in the external granular layer cell migration to internal granular layer reflected by the wide external granular layer at day 7 and day 15. Purkinje cells are arranged in more than one row and appear abnormal, small, and spindle in shape. (j–l) Sections in the cerebellum of *C. sinensis*-supplemented nicotine-induced mice newborns showing improved histological structure. Scale bar = 50 *μ*m.

The cerebellar histological sections of all groups showed the neural fold layer structures at D7, D15, and D30. In normal newborns, the external granular layer appeared wide at D7 (Figure 6(a)), thin at D15 (Figure 6(b)), and disappeared completely at D30 (Figure 6(c)). The molecular layer was defined at D7, wide at D15, and incubated by mature neurons at D30. The Purkinje cells were arranged in one row at D7 to form a Purkinje cell layer. The Purkinje cells became more developed and mature at D15 and D30. The internal granular layer received the migrated cells from the external granular layer, so it appeared condensed at the three investigated ages. The cerebellar sections of the *C. sinensis*-administered group showed well-developed cerebellar fold layers similar to those of the normal newborns (Figures 6(d), 6(e), and 6(f)). In the nicotine-exposed group, some aberrations in the cerebellar fold layers appeared including a delay in the external granular layer cell migration to internal granular layer that was reflected by the wide external granular layer at D7 (Figure 6(g)) and D15 (Figure 6(h)) when compared with the control group. Purkinje cells appeared arranged in more than one row at D7. At D15 and D30 (Figure 6(i)), Purkinje cells appeared abnormal, small, and spindle in shape. Supplementation of *C. sinensis* prevented nicotine-induced malformations in the cerebellum of mice newborns at D7 (Figure 6(j)), D15 (Figure 6(k)), and D30 (Figure 6(l)).

**Figure 7:**
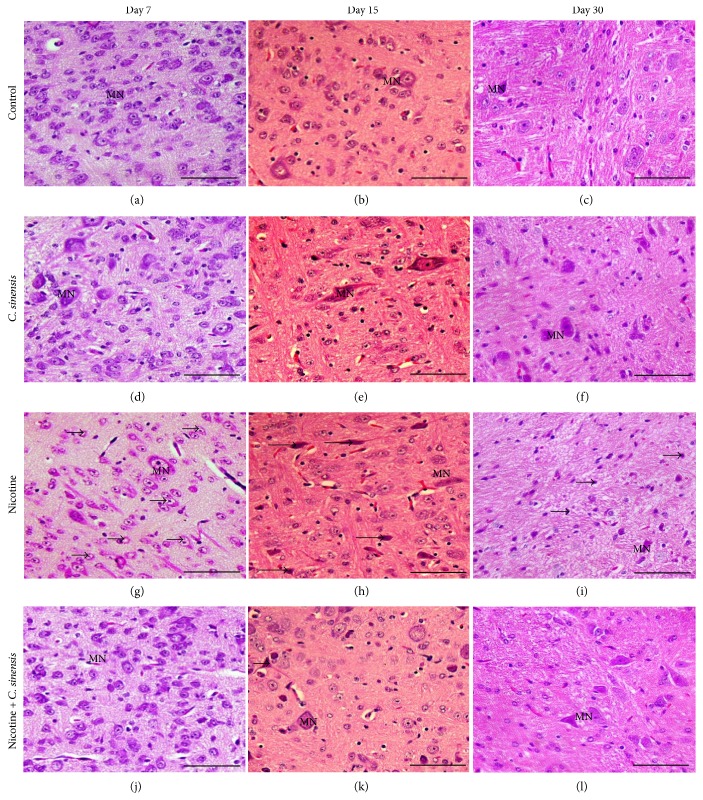
Sagittal sections in the medulla oblongata of (a–c) control newborns showing normal state and distribution of medullary neurons, (d–f) the *C. sinensis*-administered group showing normal histological structures of the medullary neurons, (g–i) the nicotine-exposed group revealing pyknosis and chromatolysis (arrow) of the medullary neurons (MN), and (j–l) the *C. sinensis*-supplemented nicotine-induced mice newborns showing normal histology of the medulla oblongata. Scale bar = 50 *μ*m.

Histological examination of the medulla oblongata sections of control newborns at D7 (Figure 7(a)), D15 (Figure 7(b)), and D30 (Figure 7(c)) showed normal state and distribution of medullary neurons. The *C. sinensis*-administered group showed normal histological structures of the medullary neurons (Figures 7(d), 7(e), and 7(f)). Perinatal nicotine exposure induced pyknosis and chromatolysis of the medullary neurons at D7 (Figure 7(g)), D15 (Figure 7(h)), and D30 (Figure 7(i)). Nicotine-induced mice newborns treated with *C. sinensis* showed normal histology of the medulla oblongata at D7 (Figure 7(j)), D15 (Figure 7(k)), and D30 (Figure 7(l)). Similar findings were observed in both male and female mice offspring.

### 3.4. *C. sinensis* Attenuates Nicotine-Induced Oxidative Stress in the Cerebellum and Medulla Oblongata of Mice Newborns

Lipid peroxidation, GSH, and SOD were determined to evaluate the protective effect of *C. sinensis* against nicotine-induced oxidative stress in the cerebellum and medulla oblongata of newborn mice.

**Figure 8:**
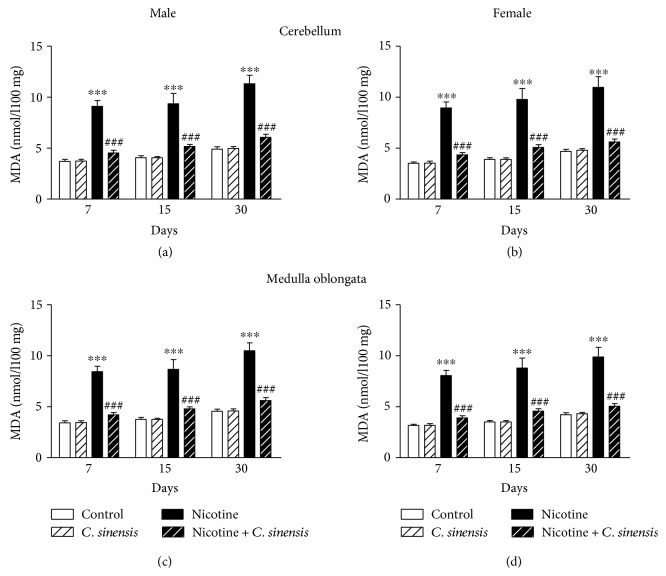
*C. sinensis* protects against nicotine-induced lipid peroxidation in the cerebellum and medulla oblongata of mice newborns. Data are M ± SEM. ^∗∗∗^*P* < 0.001 versus control and ^###^*P* < 0.001 versus nicotine.

Cerebellar lipid peroxidation showed a significant (*P* < 0.001) increase in nicotine-induced male (Figure 8(a)) and female (Figure 8(b)) mice newborns at D7, D15, and D30 after birth, an effect that was significantly (*P* < 0.001) prevented by *C. sinensis*. In the cerebellum of control male and female mice offspring, supplementation of *C. sinensis* produced nonsignificant (*P* < 0.05) effect on lipid peroxidation levels at all experimental periods.

In the medulla oblongata, nicotine induced a significant (*P* < 0.001) increase in lipid peroxidation levels in both male (Figure 8(c)) and female (Figure 8(d)) mice offspring. Oral supplementation of *C. sinensis* extract significantly (*P* < 0.001) decreased lipid peroxidation in the medulla oblongata of nicotine-induced both male and female newborn mice at D7, D15, and D30. *C. sinensis* extract produced nonsignificant (*P* > 0.05) effects on lipid peroxidation levels in the medulla oblongata of control mice offspring.

**Figure 9:**
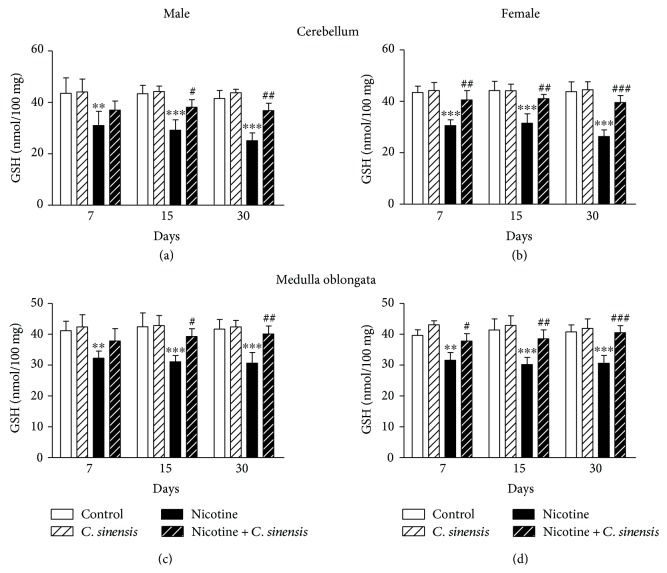
*C. sinensis* alleviates GSH levels in the cerebellum and medulla oblongata of nicotine-induced mice newborns. Data are M ± SEM. ^∗∗^*P* < 0.01 and ^∗∗∗^*P* < 0.001 versus control. ^#^*P* < 0.05, ^##^*P* < 0.01, and ^###^*P* < 0.001 versus nicotine.

GSH content in the cerebellum of nicotine-induced male newborns showed significant decrease at D7 (*P* < 0.01), D15 (*P* < 0.001), and D30 (*P* < 0.001) after birth (Figure 9(a)). Oral supplementation of *C. sinensis* extract during pregnancy exerted nonsignificant (*P* > 0.05) effect on GSH levels at D7 while producing a significant increase at D15 (*P* < 0.05) and D30 (*P* < 0.01) in the cerebellum of nicotine-induced male offspring. Nicotine-induced female mice newborns exhibited a significant (*P* < 0.001) decrease in the cerebellar GSH levels at D7, D15, and D30 after birth (Figure 9(b)). *C. sinensis* ameliorated the cerebellar GSH levels in nicotine-induced mice newborns at D7 (*P* < 0.01), D15 (*P* < 0.01), and D30 (*P* < 0.001) after birth.

Nicotine-induced male and female mice offspring showed significant decrease in medulla oblongata GSH levels at D7 (*P* < 0.01), D15 (*P* < 0.001), and D30 (*P* < 0.001) after birth. *C. sinensis* significantly ameliorated GSH levels in the medulla oblongata of nicotine-induced male mice offspring at D15 (*P* < 0.05) and D30 (*P* < 0.01); however, its effect at D7 was nonsignificant (Figure 9(c)). In nicotine-induced female mice newborns, *C. sinensis* supplementation significantly increased GSH levels in the medulla oblongata at D7 (*P* < 0.05), D15 (*P* < 0.01), and D30 (*P* < 0.001) after birth (Figure 9(d)).

*C. sinensis* exerted nonsignificant effect on the levels of GSH in the cerebellum and medulla oblongata of both control male and female mice newborn at all experimental periods.

**Figure 10:**
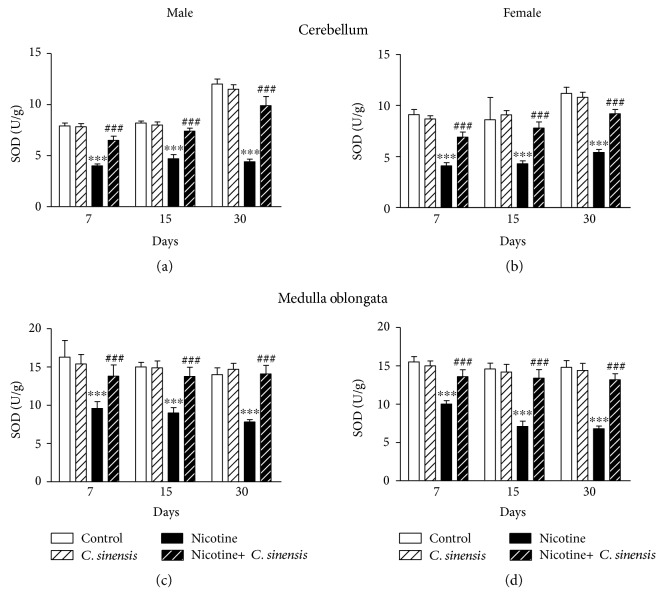
*C. sinensis* enhances SOD activity in the cerebellum and medulla oblongata of nicotine-induced mice newborns. Data are M ± SEM. ^∗∗∗^*P* < 0.001 versus control and ^###^*P* < 0.001 versus nicotine.

SOD activity in the cerebellum of nicotine-induced male (Figure 10(a)) and female (Figure 10(b)) mice offspring showed a significant (*P* < 0.001) decrease at D7, D15, and D30 after birth, an effect that was significantly (*P* < 0.001) prevented by *C. sinensis*. In the cerebellum of control male and female mice offspring, *C. sinensis* exerted nonsignificant (*P* > 0.05) effect on SOD activity at all experimental periods.

In the medulla oblongata, nicotine induced a significant (*P* < 0.001) decline in SOD activity in both male (Figure 10(c)) and female (Figure 10(d)) mice offspring. Oral supplementation of *C. sinensis* extract significantly (*P* < 0.001) improved SOD activity in the medulla oblongata of both male and female newborn mice at D7, D15, and D30 while exerting nonsignificant (*P* > 0.05) effect in control mice offspring.

## 4. Discussion

Cigarette smoking is a common problem and 10–15% women continue smoking during pregnancy even with the well-known detrimental outcomes in newborns [12, 45, 46]. Because of the dynamic nature of developmental processes, the central nervous system is vulnerable to damage by environmental toxins during fetal and early postnatal life [45, 47]. Abnormal behaviors in offspring have been associated with maternal smoking during pregnancy. These neurodevelopmental adverse effects might extend through adolescence and adulthood [12, 46]. In the present study, we demonstrated the potential protective effect of *C. sinensis* against perinatal nicotine-induced neurodevelopmental alterations in male and female offspring.

Male and female offspring born to mice exposed to nicotine during pregnancy and lactation showed declined body weight and a delay in eyes opening and body hair appearance, indicating the adverse effects of nicotine on the physical growth in mice offspring. Maternal exposure to nicotine during pregnancy has been associated with reduced fat index in monkeys [48] and fetal body weight in rats [49, 50]. The declined body weight could be also explained, at least in part, by the increased lipolysis [51], energy expenditure, and metabolic rate [52]. These experimental data were supported by the clinical findings where infants, exposed to nicotine during the third trimester, showed smaller body weight at birth [18, 53]. Recently, Chan et al. [54] reported decreased body weight of male mice born to mice exposed to cigarette smoke. Oral supplementation of *C. sinensis* significantly prevented nicotine-induced in utero growth retardation. This physical improvement could be explained in terms of improved energy homeostasis by *C. sinensis*.

Nicotine and its metabolite cotinine pass through the placenta and bind to the nicotinic acetylcholine receptors [7], which are expressed by the second gestational week in rodents and in the first trimester in humans [8, 9]. Studies have demonstrated that the developmental exposure to nicotine disturbs the cholinergic system and therefore disrupts brain development, neuronal migration, synaptogenesis, and neurotransmitter release [11]. These adverse effects might produce neurobehavioral impairments in the offspring.

Here, perinatal exposure to nicotine markedly affected the motor development, active avoidance response, and locomotory behavior. The righting, rotating, and cliff avoidance reflexes were significantly suppressed in male and female pups born to mothers exposed to nicotine. These findings suggest a direct impact of nicotine exposure in utero and during lactation period on the neuromotor development.

Nicotine-induced male and female mice newborns showed declined memory and learning ability as measured by the shuttle-box test. Additionally, the mice took longer time exploring the closed arm while spending short time to explore the open arm, therefore, showing increased anxiety-like behavior. Furthermore, nicotine-exposed male and female mice showed suppressed locomotory activity and equilibrium reflex.

In line with our findings, the study of Khalki et al. [14] demonstrated delayed development of similar behavioral patterns in rats exposed to tobacco alkaloid extracts. Studies have also reported poor performance of memory and learning test, including two-way active avoidance [16] and the radial arm maze [15, 55] in rodents exposed to nicotine in utero. Perinatal exposure to nicotine can also lead to spatial memory deficits [56]. Mice in the radial arm maze took longer time to reach the criterion when injected with nicotine [57]. Moreover, mice exposed to nicotine exhibited sensory processing defects evidenced by hypersensitive passive avoidance [17]. These experimental data were in agreement with multiple clinical findings where exposure to nicotine in utero induced several alterations, including poor attention, increased tremors, hypertonicity, startle responses, irritability, and deficient speech processing in newborns [18–21]. In addition, infants exposed to nicotine in utero showed an attenuated response to auditory stimuli which, later in life, can possibly lead to language and learning impairments [20, 22].

Interestingly, supplementation of *C. sinensis* to mice markedly prevented nicotine-induced neurobehavioral alterations. *C. sinensis* significantly improved motor development, active avoidance responses, locomotory behavior, equilibrium reflex, and memory and learning ability.

We thought that the protective effects of *C. sinensis* extract originate from its ability to counteract nicotine-induced tissue damage and oxidative stress. Nonhuman primates exposed to tobacco smoke during gestation and lactation exhibited neuronal cell loss and decreased cell size [58]. Juvenile and adult rats exposed to nicotine in utero showed changes in dendritic length and dendritic branching [59]. Pauly and Slotkin [60] stated that these changes can alter the development and contribute to functional deficits later in life. The study of Ernst et al. [61] attributed impaired cognitive function in prenatal nicotine-exposed rats to a disruption of neuronal migration in the brain. In the present study, nicotine induced a delay in cell migration from the external granular layer to the internal layer and altered shape and pyknosis in Purkinje cells in the cerebellum and chromatolysis of neurons in the medulla oblongata, an effect that was markedly prevented in the *C. sinensis*-supplemented groups. These findings highlight the efficacy of *C. sinensis* in preventing cell damage induced by nicotine.

Attenuation of oxidative stress and alleviation of the antioxidant defenses is another mechanism we hypothesized to contribute to the protective effect of *C. sinensis* on nicotine-induced neurodevelopmental and behavioral changes in mice newborn. Our hypothesis is supported by findings of multiple studies demonstrating alleviated neurobehavioral performance in experimental animals supplemented with antioxidants [30, 62]. Previous studies have attributed memory impairment following prenatal nicotine exposure to excessive production of ROS and its subsequent cell death in various brain regions [28–30]. Because the cerebellum is involved in motor control, cognitive functions, and regulating fear and pleasure and the medulla oblongata is responsible for the regulation of reflexes, we assayed lipid peroxidation and antioxidant defenses in these brain regions. Exposure to nicotine increased lipid peroxidation in the cerebellum and medulla oblongata of both male and female newborns as evidenced by the elevated levels of MDA. On the other hand, GSH and SOD were significantly declined in the cerebellum and medulla oblongata of male and female newborns. These alterations were observed at D7, D15, and D30 after birth. Brain neurons are sensitive to oxidative stress [63], and maternal nicotine and cigarette smoke exposure has been confirmed to induce oxidative stress in the brain of offspring. Chan et al. [54] demonstrated increased oxidative stress and declined antioxidant defenses in the brain of mice offspring whose mothers were exposed to cigarette smoke during pregnancy. Recently, we reported increased lipid peroxidation and declined antioxidants in the cerebrum of offspring born to mice exposed to nicotine during gestation and early period of lactation [26].

*C. sinensis* supplementation during pregnancy and early postnatal period significantly prevented nicotine-induced lipid peroxidation and improved the antioxidant defenses in the cerebellum and medulla oblongata of both male and female newborns. Therefore, we assumed that the antioxidant potential of *C. sinensis* protected the newborns against nicotine-induced oxidative stress, cell death, and neurodevelopmental alterations. The antioxidant efficacy of *C. sinensis* and its active constituents such as polyphenols has been well-established in several studies [64, 65]. Polyphenols of *C. sinensis* prevented 6-hydroxydopamine-induced damage of dopaminergic neurons in a rat model of Parkinson's disease [66]. Epigallocatechin-3-gallate (EGCG), a major bioactive catechin of *C. sinensis*, protected the rat cortex against acrylamide-induced apoptosis and astrogliosis [64] and oxidative stress in PC12 cells [65]. The neuroprotective effect induced by catechins is thought to occur through potentiation of antioxidant defenses [67], activation of protein kinase C, and upregulation of cell survival genes [68].

In summary, the current findings show that *C. sinensis* confers protective effect against nicotine-induced neurobehavioral alterations and oxidative stress. Perinatal nicotine exposure induced potential neurotoxic effects evidenced by chromatolysis of neurons, increased ROS production and neurobehavioral alterations in male and female newborns. *C. sinensis* significantly protected male and female newborns against nicotine-induced neurotoxicity. Therefore, *C. sinensis* represents a potential candidate conferring protection against nicotine-induced neurotoxicity in offspring, pending further studies to determine the exact mechanisms of action.

## Figures and Tables

**Table 1:** Effect of perinatal nicotine exposure and *C. sinensis* extract on the locomotor activity of mice newborns at adolescent age (postnatal day 22).

		Control	*C. sinensis*	Nicotine	Nicotine + *C. sinensis*
Number of squares crossed	Male	210.5 (170–230)	303^∗^ (193–320)	110^∗∗∗^ (99–133)	157^∗∗^^#^ (153–170)
	Female	190.5 (170–211)	289^∗^ (188–310)	112^∗∗∗^ (87–123)	145^∗∗^^#^ (134–165)
Wall rears	Male	20.5 (15–22)	27^∗^ (21–31)	11^∗∗∗^ (10–15)	9^∗∗^^#^ (1–16)
	Female	19 (10–25)	25^∗^ (22–36)	10^∗∗∗^ (9–14)	9^∗∗^^#^ (1–10)
Rears	Male	6 (0–14)	8^∗^ (3–13)	4 (1–4)	5 (1–6)
	Female	6 (1–11)	9^∗^ (1–14)	6 (3–11)	5 (1–11)
Wash	Male	5 (5–25)	11 (5–15)	15^∗^ (11–19)	6 (1–14)
	Female	12 (3–14)	13 (4–19)	16 (10–20)	8 (6–11)
Locomotion duration (sec)	Male	202 (173–268)	274^∗^ (261–291)	98^∗∗∗^ (77–132)	167^∗^^#^ (120–187)
	Female	189 (153–256)	263^∗^ (254–282)	105^∗∗∗^ (89–122)	154^∗^^#^ (110–187)
Immobility duration (sec)	Male	98 (41–127)	26^∗^ (9–39)	140.5^∗∗^ (101–188)	131^∗∗^^#^ (100–166)
	Female	102 (55–121)	33^∗^ (11–44)	122^∗∗^ (119–176)	143^∗∗^^#^ (106–153)

Date are M ± SEM. ^∗^*P* < 0.05, ^∗∗^*P* < 0.01, and ^∗∗∗^*P* < 0.001 versus control, and ^#^*P* < 0.05 versus nicotine.

## References

[1] Chahal N., McLain A. C., Ghassabian A. (2016). Maternal smoking and newborn cytokine and immunoglobulin levels. *Nicotine and Tobacco Research*.

[2] Higgins S. (2002). Smoking in pregnancy. *Current Opinion in Obstetrics and Gynecology*.

[3] Ng S. P., Zelikoff J. T. (2007). Smoking during pregnancy: subsequent effects on offspring immune competence and disease vulnerability in later life. *Reproductive Toxicology*.

[4] Hylkema M. N., Blacquiere M. J. (2009). Intrauterine effects of maternal smoking on sensitization, asthma, and chronic obstructive pulmonary disease. *Proceedings of the American Thoracic Society*.

[5] Suarez L., Felkner M., Brender J. D., Canfield M., Hendricks K. (2008). Maternal exposures to cigarette smoke, alcohol, and street drugs and neural tube defect occurrence in offspring. *Maternal and Child Health Journal*.

[6] Dalgic A., Armagan E., Helvacioglu F. (2009). High dose cotinine may induce neural tube defects in a chick embryo model. *Turkish Neurosurgery*.

[7] Lambers D. S., Clark K. E. (1996). The maternal and fetal physiologic effects of nicotine. *Seminars in Perinatology*.

[8] Dwyer J. B., Broide R. S., Leslie F. M. (2008). Nicotine and brain development. *Birth Defects Research Part C: Embryo Today*.

[9] Dwyer J. B., McQuown S. C., Leslie F. M. (2009). The dynamic effects of nicotine on the developing brain. *Pharmacology and Therapeutics*.

[10] Berlin I., Heilbronner C., Georgieu S., Meier C., Spreux-Varoquaux O. (2010). Newborns’ cord blood plasma cotinine concentrations are similar to that of their delivering smoking mothers. *Drug and Alcohol Dependence*.

[11] Slotkin T. A. (2004). Cholinergic systems in brain development and disruption by neurotoxicants: nicotine, environmental tobacco smoke, organophosphates. *Toxicology and Applied Pharmacology*.

[12] Cornelius M. D., Goldschmidt L., Day N. L. (2012). Prenatal cigarette smoking: long-term effects on young adult behavior problems and smoking behavior. *Neurotoxicology and Teratology*.

[13] Cornelius M. D., Goldschmidt L., De Genna N. M., Larkby C. (2012). Long-term effects of prenatal cigarette smoke exposure on behavior dysregulation among 14-year-old offspring of teenage mothers. *Maternal and Child Health Journal*.

[14] Khalki H., Khalki L., Aboufatima R. (2012). Prenatal exposure to tobacco extract containing nicotinic alkaloids produces morphological and behavioral changes in newborn rats. *Pharmacology Biochemistry and Behavior*.

[15] Levin E. D., Briggs S. J., Christopher N. C., Rose J. E. (1993). Prenatal nicotine exposure and cognitive performance in rats. *Neurotoxicology and Teratology*.

[16] Vaglenova J., Parameshwaran K., Suppiramaniam V., Breese C. R., Pandiella N., Birru S. (2008). Long-lasting teratogenic effects of nicotine on cognition: gender specificity and role of AMPA receptor function. *Neurobiology of Learning and Memory*.

[17] Heath C. J., King S. L., Gotti C., Marks M. J., Picciotto M. R. (2010). Cortico-thalamic connectivity is vulnerable to nicotine exposure during early postnatal development through alpha4/beta2/alpha5 nicotinic acetylcholine receptors. *Neuropsychopharmacology*.

[18] Espy K. A., Fang H., Johnson C., Stopp C., Wiebe S. A. (2011). Prenatal tobacco exposure: developmental outcomes in the neonatal period. *Developmental Psychology*.

[19] Key A. P., Ferguson M., Molfese D. L., Peach K., Lehman C., Molfese V. J. (2007). Smoking during pregnancy affects speech-processing ability in newborn infants. *Environmental Health Perspectives*.

[20] Mansi G., Raimondi F., Pichini S. (2007). Neonatal urinary cotinine correlates with behavioral alterations in newborns prenatally exposed to tobacco smoke. *Pediatric Research*.

[21] Stroud L. R., Paster R. L., Goodwin M. S. (2009). Maternal smoking during pregnancy and neonatal behavior: a large-scale community study. *Pediatrics*.

[22] Kable J. A., Coles C. D., Lynch M. E., Carroll J. (2009). The impact of maternal smoking on fast auditory brainstem responses. *Neurotoxicology and Teratology*.

[23] Stroud L. R., Paster R. L., Papandonatos G. D. (2009). Maternal smoking during pregnancy and newborn neurobehavior: effects at 10 to 27 days. *Journal of Pediatrics*.

[24] Lin C., Yon J. M., Hong J. T. (2014). 4-O-methylhonokiol inhibits serious embryo anomalies caused by nicotine via modulations of oxidative stress, apoptosis, and inflammation. *Birth Defects Research Part B: Developmental and Reproductive Toxicology*.

[25] Sudheer A. R., Chandran K., Marimuthu S., Menon V. P. (2005). Ferulic acid modulates altered lipid profiles and prooxidant/antioxidant status in circulation during nicotine-induced toxicity: a dose-dependent study. *Toxicology Mechanisms and Methods*.

[26] Al-Basher G., Ajarem J. S., Allam A. A., Mahmoud A. M. (2017). Green tea protects against perinatal nicotine-induced histological, biochemical and hematological alterations in mice offspring. *International Journal of Pharmacology*.

[27] Sudheer A. R., Muthukumaran S., Devipriya N., Menon V. P. (2007). Ellagic acid, a natural polyphenol protects rat peripheral blood lymphocytes against nicotine-induced cellular and DNA damage in vitro: with the comparison of N-acetylcysteine. *Toxicology*.

[28] Guan Z. Z., Yu W. F., Nordberg A. (2003). Dual effects of nicotine on oxidative stress and neuroprotection in PC12 cells. *Neurochemistry International*.

[29] Slotkin T. A., Seidler F. J., Qiao D. (2005). Effects of prenatal nicotine exposure on primate brain development and attempted amelioration with supplemental choline or vitamin C: neurotransmitter receptors, cell signaling and cell development biomarkers in fetal brain regions of rhesus monkeys. *Neuropsychopharmacology*.

[30] Sirasanagandla S. R., Rooben R. K., Rajkumar, Narayanan S. N., Jetti R. (2014). Ascorbic acid ameliorates nicotine exposure induced impaired spatial memory performances in rats. *West Indian Medical Journal*.

[31] Butt M. S., Ahmad R. S., Sultan M. T., Qayyum M. M., Naz A. (2015). Green tea and anticancer perspectives: updates from last decade. *Critical Reviews in Food Science and Nutrition*.

[32] Ghafurniyan H., Azarnia M., Nabiuni M., Karimzadeh L. (2015). The effect of green tea extract on reproductive improvement in estradiol valerate-induced polycystic ovarian syndrome in rat. *Iranian Journal of Pharmaceutical Research*.

[33] Umezu T. (2012). Unusual effects of nicotine as a psychostimulant on ambulatory activity in mice. *ISRN Pharmacology*.

[34] Ajarem J. S., Ahmad M. (1996). Teratopharmacological and behavioral effects of coffee in mice. *Acta Physiologica et Pharmacologica Bulgarica*.

[35] Ajarem J. S., Ahmad M. (1991). Behavioral and biochemical consequences of perinatal exposure of mice to instant coffee: a correlative evaluation. *Pharmacology Biochemistry and Behavior*.

[36] Ajarem J. S. (2000). Effects of prenatal nicotine exposure on some morphological, behavioral and enzymatic characteristics in mice offspring. *Journal of Union of Arab Biologists*.

[37] Ajarem J. S., Ahmad M. (1998). Prenatal nicotine exposure modifies behavior of mice through early development. *Pharmacology Biochemistry and Behavior*.

[38] Abu-Taweel G. M. (2012). Effects of perinatal exposure of lithium on neuro-behaviour of developing mice offspring. *Indian Journal of Experimental Biology*.

[39] Allam A. A., Maodaa S. N., Abo-Eleneen R., Ajarem J. (2016). Protective effect of parsley juice (Petroselinum crispum, Apiaceae) against cadmium deleterious changes in the developed albino mice newborns (Mus musculus) brain. *Oxididative Medicine and Cellular Longevity*.

[40] Abu-Taweel G. M., ZM A., Ajarem J. S., Ahmad M. (2014). Cognitive and biochemical effects of monosodium glutamate and aspartame, administered individually and in combination in male albino mice. *Neurotoxicology and Teratology*.

[41] Preuss H. G., Jarrell S. T., Scheckenbach R., Lieberman S., Anderson R. A. (1998). Comparative effects of chromium, vanadium and Gymnema sylvestre on sugar-induced blood pressure elevations in SHR. *Journal of the American College of Nutrition*.

[42] Beutler E., Duron O., Kelly B. M. (1963). Improved method for the determination of blood glutathione. *The Journal of Laboratory and Clinical Medicine*.

[43] Marklund S., Marklund G. (1974). Involvement of the superoxide anion radical in the autoxidation of pyrogallol and a convenient assay for superoxide dismutase. *European Journal of Biochemistry*.

[44] Wall P. M., Messier C. (2001). Methodological and conceptual issues in the use of the elevated plus-maze as a psychological measurement instrument of animal anxiety-like behavior. *Neuroscience and Biobehavioral Reviews*.

[45] Amos-Kroohs R. M., Williams M. T., Braun A. A. (2013). Neurobehavioral phenotype of C57BL/6J mice prenatally and neonatally exposed to cigarette smoke. *Neurotoxicology and Teratology*.

[46] Elmasry H., Goodwin R. D., Terry M. B., Tehranifar P. (2014). Early life exposure to cigarette smoke and depressive symptoms among women in midlife. *Nicotine and Tobacco Research*.

[47] Balsevich G., Poon A., Goldowitz D., Wilking J. A. (2014). The effects of pre- and post-natal nicotine exposure and genetic background on the striatum and behavioral phenotypes in the mouse. *Behavioural Brain Research*.

[48] Grove K. L., Sekhon H. S., Brogan R. S., Keller J. A., Smith M. S., Spindel E. R. (2001). Chronic maternal nicotine exposure alters neuronal systems in the arcuate nucleus that regulate feeding behavior in the newborn rhesus macaque. *Journal of Clinical Endocrinology and Metabolism*.

[49] Paccola C. C., Neves F. M., Cipriano I., Stumpp T., Miraglia S. M. (2014). Effects of prenatal and lactation nicotine exposure on rat testicular interstitial tissue. *Andrology*.

[50] Mao C., Yuan X., Zhang H. (2008). The effect of prenatal nicotine on mRNA of central cholinergic markers and hematological parameters in rat fetuses. *International Journal of Developmental Neuroscience*.

[51] Hellerstein M. K., Benowitz N. L., Neese R. A. (1994). Effects of cigarette smoking and its cessation on lipid metabolism and energy expenditure in heavy smokers. *Journal of Clinical Investigation*.

[52] Nunn J. F. (1993). Chapter 19 - smoking. *Nunn’s Applied Respiratory Physiology*.

[53] Himes S. K., Stroud L. R., Scheidweiler K. B., Niaura R. S., Huestis M. A. (2013). Prenatal tobacco exposure, biomarkers for tobacco in meconium, and neonatal growth outcomes. *Journal of Pediatrics*.

[54] Chan Y. L., Saad S., Pollock C. (2016). Impact of maternal cigarette smoke exposure on brain inflammation and oxidative stress in male mice offspring. *Scientific Reports*.

[55] Sorenson C. A., Raskin L. A., Suh Y. (1991). The effects of prenatal nicotine on radial-arm maze performance in rats. *Pharmacology Biochemistry and Behavior*.

[56] Eppolito A. K., Smith R. F. (2006). Long-term behavioral and developmental consequences of pre- and perinatal nicotine. *Pharmacology Biochemistry and Behavior*.

[57] Yanai J., Pick C. G., Rogel-Fuchs Y., Zahalka E. A. (1992). Alterations in hippocampal cholinergic receptors and hippocampal behaviors after early exposure to nicotine. *Brain Research Bulletin*.

[58] Slotkin T. A., Pinkerton K. E., Seidler F. J. (2006). Perinatal environmental tobacco smoke exposure in rhesus monkeys: critical periods and regional selectivity for effects on brain cell development and lipid peroxidation. *Environmental Health Perspectives*.

[59] Muhammad A., Mychasiuk R., Nakahashi A., Hossain S. R., Gibb R., Kolb B. (2012). Prenatal nicotine exposure alters neuroanatomical organization of the developing brain. *Synapse*.

[60] Pauly J. R., Slotkin T. A. (2008). Maternal tobacco smoking, nicotine replacement and neurobehavioural development. *Acta Paediatrica*.

[61] Ernst M., Moolchan E. T., Robinson M. L. (2001). Behavioral and neural consequences of prenatal exposure to nicotine. *Journal of the American Academy of Child and Adolescent Psychiatry*.

[62] Narayanan S. N., Kumar R. S., Paval J., Nayak S. (2010). Effect of ascorbic acid on the monosodium glutamate-induced neurobehavioral changes in periadolescent rats. *Bratislava Medical Journal*.

[63] Urrutia P. J., Mena N. P., Nunez M. T. (2014). The interplay between iron accumulation, mitochondrial dysfunction, and inflammation during the execution step of neurodegenerative disorders. *Frontiers in Pharmacology*.

[64] He Y., Tan D., Bai B., Wu Z., Ji S. (2017). Epigallocatechin-3-gallate attenuates acrylamide-induced apoptosis and astrogliosis in rat cerebral cortex. *Toxicology Mechanisms and Methods*.

[65] He Y., Tan D., Mi Y. (2016). Effect of epigallocatechin-3-gallate on acrylamide-induced oxidative stress and apoptosis in PC12 cells. *Human and Experimental Toxicology*.

[66] Guo S., Yan J., Yang T., Yang X., Bezard E., Zhao B. (2007). Protective effects of green tea polyphenols in the 6-OHDA rat model of Parkinson’s disease through inhibition of ROS-NO pathway. *Biological Psychiatry*.

[67] Al-Malki A. L., Moselhy S. S. (2013). Protective effect of vitamin E and epicatechin against nicotine-induced oxidative stress in rats. *Toxicology and Industrial Health*.

[68] Mandel S. A., Avramovich-Tirosh Y., Reznichenko L. (2005). Multifunctional activities of green tea catechins in neuroprotection. Modulation of cell survival genes, iron-dependent oxidative stress and PKC signaling pathway. *Neurosignals*.

